# The TB27 Transcriptomic Model for Predicting *Mycobacterium tuberculosis* Culture Conversion

**DOI:** 10.20411/pai.v10i1.770

**Published:** 2025-01-29

**Authors:** Maja Reimann, Korkut Avsar, Andrew R. DiNardo, Torsten Goldmann, Gunar Günther, Michael Hoelscher, Elmira Ibraim, Barbara Kalsdorf, Stefan H.E. Kaufmann, Niklas Köhler, Anna M. Mandalakas, Florian P. Maurer, Marius Müller, Dörte Nitschkowski, Ioana D. Olaru, Cristina Popa, Andrea Rachow, Thierry Rolling, Helmut J. F. Salzer, Patricia Sanchez-Carballo, Maren Schuhmann, Dagmar Schaub, Victor Spinu, Elena Terhalle, Markus Unnewehr, Nika J. Zielinski, Jan Heyckendorf, Christoph Lange

**Affiliations:** 1 Clinical Infectious Diseases, Research Center Borstel, Borstel, Germany; 2 German Center for Infection Research (DZIF), Partner Site Hamburg-Lübeck-Borstel-Riems, Germany; 3 Respiratory Medicine & International Health, University of Lübeck, Lübeck, Germany; 4 Asklepios Fachkliniken München-Gauting, Munich, Germany; 5 The Global Tuberculosis Program, Texas Children's Hospital, Immigrant and Global Health, Department of Pediatrics, Baylor College of Medicine, Houston, Texas; 6 Department of Internal Medicine and Radboud Center for Infectious Diseases, Radboud University Medical Center, Nijmegen, The Netherlands; 7 Division of Histopathology, Research Center Borstel, Leibniz Lung Center, Borstel, Germany; 8 German Center for Lung Research (DZL), Airway Research Center North, Borstel, Germany; 9 Department of Medicine, University of Namibia School of Medicine, Windhoek, Namibia; 10 Inselspital Bern, Department of Pulmonology, Bern, Switzerland; 11 Division of Infectious Diseases and Tropical Medicine, University Hospital, LMU Munich, Munich, Germany; 12 German Center for Infection Research (DZIF), partner site Munich, Germany; 13 Fraunhofer Institute for Translational Medicine and Pharmacology ITMP; Immunology, Infection and Pandemic Research, Munich, Germany; 14 Unit Global Health, Helmholtz Zentrum München, German Research Center for Environmental Health (HMGU), Neuherberg, Germany; 15 Institutul de Pneumoftiziologie “Marius Nasta”, MDR-TB Research Department, Bucharest, Romania; 16 Max Planck Institute for Infection Biology, Berlin, Germany; 17 Max Planck Institute for Multidisciplinary Sciences, Göttingen, Germany; 18 Hagler Institute for Advanced Study, Texas A&M University, College Station, Texas; 19 Charité – Universitätsmedizin Berlin, corporate member of Freie Universität Berlin and Humboldt-Universität zu Berlin, Germany; 20 Division of Infectious Diseases, I. Department of Internal Medicine, University Medical Center Hamburg-Eppendorf, Hamburg, Germany; 21 National and WHO Supranational Reference Laboratory for Mycobacteria, Research Center Borstel, Borstel, Germany; 22 Institute of Medical Microbiology, Virology and Hygiene, University Medical Center Hamburg-Eppendorf, Hamburg, Germany; 23 new affiliation: Roche Diagnostics, Zurich, Switzerland; 24 Sankt Katharinen-Krankenhaus, Frankfurt, Germany; 25 Infektiologikum, Frankfurt, Germany; 26 London School of Hygiene and Tropical Medicine, London, United Kingdom; 27 new affiliation: Medical Microbiology, University of Münster, Germany; 28 Department of Clinical Immunology of Infectious Diseases, Bernhard-Nocht-Institute for Tropical Medicine, Hamburg, Germany; 29 new affiliation: Biontech SE, Mainz, Germany; 30 Division of Infectious Diseases and Tropical Medicine, Department of Internal Medicine 4-Pneumology, Kepler University Hospital, Linz, Austria; 31 Medical Faculty, Johannes Kepler University Linz, Linz, Austria; 32 Ignaz-Semmelweis-Institute, Interuniversity Institute for Infection Research, Vienna, Austria; 33 new affiliation: Lungenpraxis Konstanz, Konstanz, Germany; 34 new affiliation: LungenClinic Großhansdorf, Großhansdorf, Germany; 35 Department of Respiratory Medicine and Infectious Diseases, St. Barbara-Klinik, Hamm, Germany; 36 Department of Medicine, Faculty of Health, Witten/Herdecke University, Witten, Germany; 37 new affiliation: Internal Medicine II, Leibniz LungClinic, University Hospital Schleswig-Holstein (UKSH) Campus Kiel, Germany; 38 new affiliation: Pulmonology and Inflammation Medicine, Christian-Albrechts-University Kiel, Germany

**Keywords:** biomarker, therapy response, tuberculosis treatment, precision medicine, systems biology

## Abstract

**Rationale:**

Treatment monitoring of tuberculosis patients is complicated by a slow growth rate of *Mycobacterium tuberculosis*. Recently, host RNA signatures have been used to monitor the response to tuberculosis treatment.

**Objective:**

Identifying and validating a whole blood-based RNA signature model to predict microbiological treatment responses in patients on tuberculosis therapy.

**Methods:**

Using a multi-step machine learning algorithm to identify an RNA-based algorithm to predict the remaining time to culture conversion at flexible time points during anti-tuberculosis therapy.

**Results:**

The identification cohort included 149 patients split into a training and a test cohort, to develop a multistep algorithm consisting of 27 genes (TB27) for predicting the remaining time to culture conversion (TCC) at any given time. In the test dataset, predicted TCC and observed TCC achieved a correlation coefficient of *r*=0.98. An external validation cohort of 34 patients shows a correlation between predicted and observed days to TCC also of *r*=0.98.

**Conclusion:**

We identified and validated a whole blood-based RNA signature (TB27) that demonstrates an excellent agreement between predicted and observed times to *M. tuberculosis* culture conversion during tuberculosis therapy. TB27 is a potential useful biomarker for anti-tuberculosis drug development and for prediction of treatment responses in clinical practice.

## INTRODUCTION

In 2023, the World Health Organization (WHO) estimated that 10.8 million people globally developed tuberculosis (TB), and 1.2 million died from this disease [1]. Drug-susceptible (DS)-TB requires a standardized combination therapy of 4 to 6 months duration [2]. In case of bacillary resistance to rifampicin and isoniazid, defined as multidrug-resistant (MDR)-TB, or of rifampicin resistance alone (RR-TB), a combination therapy with second-line anti-TB medication over 6 months is currently recommended for the majority of affected patients [3, 4]. Regardless of the presence of *Mycobacterium tuberculosis* drug-resistance, it is essential to monitor the effect of therapy to ensure adequate treatment responses and to assess the contagiousness of patients for contacts [5, 6].

Currently, the WHO recommends TB treatment monitoring by sputum smear microscopy and/or culture after 2, 5, and 6 months [7]. Ideally, both time metrics decrease over time until microscopic acid-fast bacilli and growth of *M. tuberculosis* in culture become undetectable [8]. The detection of growth of *M. tuberculosis* in sputum culture may take weeks, and the conventional detection of acid-fast bacilli by sputum smear microscopy during anti-TB therapy cannot distinguish live from dead bacilli [8]. Therefore, a persistently positive culture after 2 months is often detected late in the course of therapy, which means that it is only possible to intervene at an advanced stage of therapy. Accuracy and predictability of both methods decline during treatment [8]. Furthermore, they cannot be applied to all patient populations, eg, young children who are unable to regularly produce sputum or patients with extrapulmonary TB [9, 10].

In early bactericidal activity studies during anti-TB drug development in Phase II trials, numbers of *M. tuberculosis* colony forming units (CFU) are taken into account [9]. However, CFU counting is labor-intensive and requires sputum with high bacterial load [10]. Although imaging techniques complement the sputum-based diagnostic and monitoring, there are no globally standardized evaluation criteria for assessing the results of imaging techniques [5].

Various pathogen-based approaches have been tested emerging as alternatives to the current standard of practice employing culture and microscopy as therapy monitoring tools. Pathogen-based examples include molecular bacterial load assays (MBLA) and lipoarabinomannan (LAM) detection [11, 12]. Both are currently not standard of care. MBLA provides significantly faster results in comparison to culture but is not suitable for later stages of therapy. Detection of LAM in urine and sputum for treatment monitoring has limited sensitivity when the bacterial load decreases [12, 13].

Host-based treatment monitoring approaches are evolving in parallel. Transcriptomic markers have been identified and validated to predict progression to TB [14], to distinguish TB patients from healthy controls [15], and to monitor treatment responses [5, 16, 17].

Until now, whole blood RNA signatures have not been correlated with bacteriological markers of anti-TB treatment responses. To address this, we aimed to identify and validate a TB-specific, host-based whole blood artificial intelligence (AI) RNA-signature biomarker algorithm. This algorithm monitors microbiological treatment response during anti-TB therapy and predicts, in a culture-free manner, the time remaining until *M. tuberculosis* culture conversion.

## MATERIALS AND METHODS

### Study Design and Participants

The recruitment of patients to the German DS Identification Cohort (DS-GIC), the German MDR Identification Cohort (MDR-GIC), the DS German Validation Cohort (DS-GVC), and the MDR German Validation Cohort (MDR-GVC) has already been described elsewhere [17]. Between October 1, 2018, and July 31, 2021, patients with DS-TB and MDR-TB were enrolled in the Second German Validation Cohort (SGVC) at the Research Center Borstel. Up to 7 (DS-TB) or up to 10 (MDR-TB) blood samples were collected at fixed time points during the course of the patients' therapy from the start of therapy to 1-year follow-up [17]. The study visits included collecting whole blood RNA from PAXgene tubes (Qiagen^®^, Venlo), clinical data such as age and gender, and culture-based data such as time to positivity (TTP), and time to culture conversion (TCC).

Details on RNA processing, labeling, hybridization, and microarray analysis, data extraction, data normalization, data analysis, free availability of RNA data, detailed steps of model development, and comparison with other published signatures or scores have been reported previously [17].

### Statistical Analysis

Patient characteristics were reported using median and interquartile range (IQR) or frequencies and percentages, and they were tested for differences between the identification and validation cohorts using Wilcoxon rank sum test, and Chi-squared or Fisher's exact test, respectively. All tests were 2-sided.

Preprocessing of microarray data was described previously [17]. Microarray data were modeled with R software (version 4.0.1) using the limma, glmnet, and MASS packages, among others. The datasets of the German cohorts DS-GIC, DS-GVC, MDR-GIC, and MDR-GVC were combined as the identification cohort and afterwards divided randomly into a training and a test dataset at a 70:30 ratio, while SGVC served as an independent external validation cohort. All datasets were z-score normalized.

In a first step, the bacterial burden score (BBS) was modeled. For this purpose, a LASSO (Least Absolute Shrinkage and Selection Operator) regression was used to identify genes that are important for the prediction of TTP. After further gene reduction steps, a generalized linear model (GLM) was created to classify the current bacterial load by training the model on TTP.

The second step was a retrodiction for the time to culture conversion (TCC) at the start of therapy. For this, only the data subset of patients at baseline was used, with TCC as the dependent variable. The genes identified by the LASSO procedure in a further GLM model were reduced to a minimum number of genes using the Akaike Information Criterion (AIC), and the days under therapy and the calculated BBS value were included in a final model for the bacterial clearance score (BCS).

In a final step, genes were identified using LASSO and subsequently AIC, which correlated with the days remaining until culture conversion at the time of blood collection.

The dependent variable was the difference between TCC and the days under therapy at the time of blood collection. These genes, days on therapy, BBS, and BCS were used in the final GLM model to predict the time remaining to culture conversion at the time of blood collection.

Bland-Altmann analysis was also used to assess the ability of the selected signatures to serve as surrogates for culture as a gold standard to provide evidence of both correlation and systematic variation.

### Performance Comparison with Existing Signatures

To further validate the discriminatory power of the model, published RNA signatures and scores were also examined for their ability to predict the remaining TCC in time and compared with the model [14, 17–30]. For this purpose, TB-associated signatures or scores with less than 100 genes were identified. The extent to which the existing signatures were able to represent any culture transformation was investigated. The genes from the signatures were used to test their respective predictive power for the remaining TCC.

Furthermore, the signatures described here and those taken from the literature were used to create random forest algorithms in the training set (70:30 ratio of the complete data set) that can distinguish between time points before and after culture conversion in the test set.

The raw probabilities from the random forest model were used and calibrated using Platt calibration [31]. The calibrated representation shows the optimal probability distribution in the test set and is compared with the actual probability distribution of the raw probabilities to show the precision of the classification of the respective raw probabilities.

## ETHICS

The study to investigate biomarkers for treatment response in patients was approved by the ethics committee of the University of Lübeck (AZ 12-233), which in turn was confirmed by the corresponding local ethics committees of all participating centers in Germany. All patients were informed about the objectives of the study and gave their broad consent to participate and to the use of clinical, microbiological, and laboratory data, which could be revoked at any time.

## RESULTS

The identification cohort consisted of 149 patients. The median age of participants in the validation cohort was 37.7 years (IQR = 26.8 - 46.9 years). Ninety-two (65.2%) patients were male and 100 (67.1%) had MDR-TB. The median baseline TTP was 15 days (IQR = 8.3 - 27.0), and the median TCC was 49 days (IQR = 22.0 - 77.5 days). In addition, 98 (77.2%) patients had cavitary disease and 68 patients (50.7%) were smokers (Table 1).

**Table 1.** Patient Characteristics of the Identification Cohort and Validation Cohort

	Identification cohort (n=149)	Validation cohort (n=34)	*P*-value
Age in years (median, IQR)	37.7 (26.8 – 46.9)	42.1 (34.3 – 53.5)	0.036*
Sex male (n, %)	92 (65.2%)	25 (75.8%)	0.341
MDR-TB (n, %)	100 (67.1%)	16 (47.1%)	0.046*
Baseline TTP in days (median, IQR)	15.0 (8.3 – 27.0)	11.5 (7.8 – 32.3)	0.621
TCC in days (median, IQR)	49.0 (22.0 – 77.5)	36.0 (21.0 – 53.0)	0.099
Cavitary disease (n, %)	98 (77.2%)	22 (68.8%)	0.448
Smoking (n, %)	68 (50.7%)	16 (48.5%)	0.969

IQR, Interquartile range; MDR-TB, multidrug-resistant tuberculosis; TTP, time to positivity; TCC, time to culture conversion

**Figure 1.**
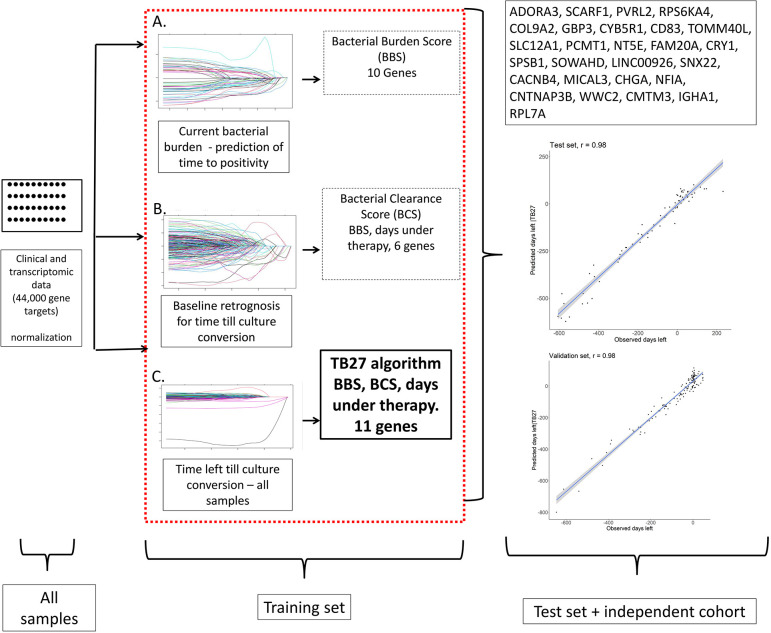
**Multi-step development for predicting time left until culture conversion.** Simplified flow chart showing the multi-step approach of transcriptomic and clinical data analysis to develop the TB27 model for predicting days left until culture conversion at any given time during therapy. Step 1: A) “Bacterial burden score (BBS)” modeling. Identification of genes using LASSO regression Genes identified for predicting time to culture positivity. After further gene reduction steps, a model consisting of 10 genes was created to draw conclusions about bacterial burden through TTP prediction. Step 2: B) Bacterial clearance score (BCS). Retro prediction for time to culture conversion at therapy initiation. Through the LASSO and other reduction procedures, 6 genes were identified to predict the time to culture conversion at therapy start. Step 3: C) TB27 Score. BBS, BCS, and 44,000 transcripts formed the basis. Gene reduction led to the final model consisting of BBS, BCS, time under therapy, and 11, which is expected to predict the remaining time to culture conversion. The model fit is R=0.98; in the test set, the correlation coefficient is *r*=0.98; in the validation set, the correlation coefficient is *r*=0.98

Figure 1 shows the modeling process and associated evaluation of the microarray datasets within 3 steps to identify and validate an RNA-based biomarker model. The identification cohort was divided in a 70:30 ratio into a training and a test set. After the model, containing 27 transcripts, was defined in the training data set and achieved a coefficient of determination of *r*²=0.98, the AI algorithm was first applied to the test data set. Here, a correlation coefficient of *r*=0.98 was found between the observed and the remaining days until culture conversion (Figure 1). The median difference between the observed and calculated remaining time to culture conversion was 1.7 days with an IQR of -4.9 to 11.6.

**Figure 2.**
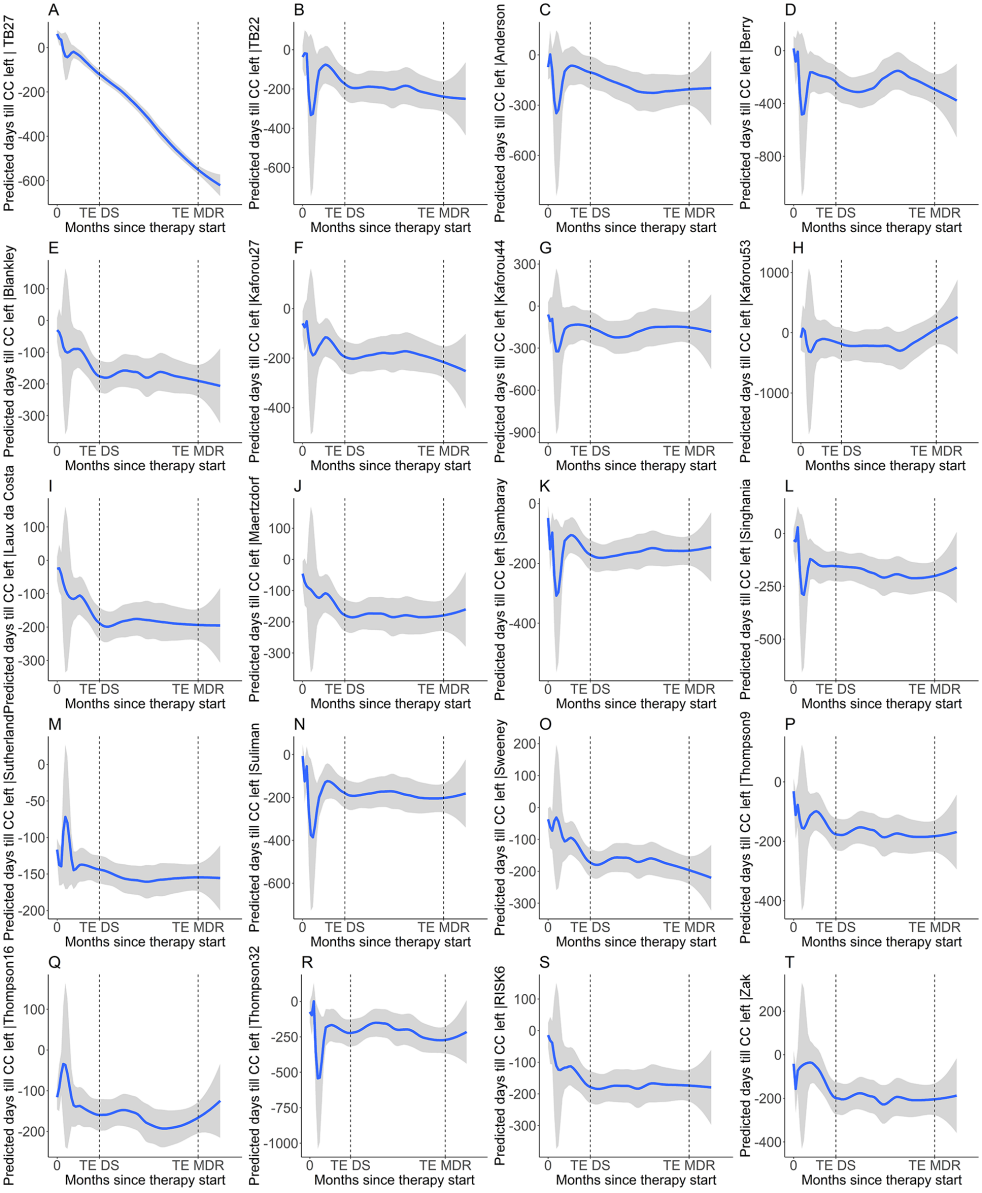
**Comparison of the 27-RNA gene signature model with published RNA-signatures and scores for predicting time left until culture conversion during therapy.** Y-axis: Predicted days left until culture conversion; X-axis: time under therapy in months. Figure 2A: TB27. Figure 2B: TB22 [17]. Figure 2C: Anderson et al, 43 genes [18]. Figure 2D: Berry et al, 87 genes [19]. Figure 2E: Blankley et al, 4 genes [20]. Figure 2F: Kaforou et al, 27 genes [21]. Figure 2G: Kaforou et al, 44 genes [21]. Figure 2H: Kaforou et al, 53 genes [21]. Figure 2I: Laux da Costa et al, 3 genes [22]. Figure 2J: Maertzdorf et al, 3 genes. Figure 2K: Sambarey et al, 10 genes [25] Figure 2L: Singhania et al, 20 genes [26]. Figure 2M: Sutherland et al, 4 genes [28]. Figure 2N: Suliman et al, 4 genes [27]. Figure 2O: Sweeney et al, 3 genes [30]. Figure 2P: Thompson et al, 9 genes [29]. Figure 2Q: Thompson et al, 16 genes [29]. Figure 2R: Thompson et al, 32 genes [29]. Figure 2S: RISK6 genes [23]. Figure 2T: Zak et al, 16 genes [14].

The independent validation cohort included 34 patients with a median age of 42.1 years (IQR = 34.3 - 53.5 years). Of the 34 patients, 25 (75.8%) were male (*P*=0.341), 16 (47.1%) had MDR-TB, 22 (68.8%) had cavitary disease (*P*=0.448), and 16 (48.5%) were active smokers (*P*=0.969). Median baseline TTP in the validation cohort was 11.5 days (IQR = 7.8 - 32.3, *P*=0.621) and TCC was reached at a median of 36 days with an IQR of 21.0 to 53.0 days (*P*=0.099) (Table 1). The AI algorithm was applied to the validation cohort, revealing a strong correlation of *r*=0.98 between the observed and predicted remaining TCC. The median difference between observed and calculated remaining TCC was 7.4 with an IQR of -8.7 to 32.5 (Figure 1, Figure 2).

**Figure 3.**
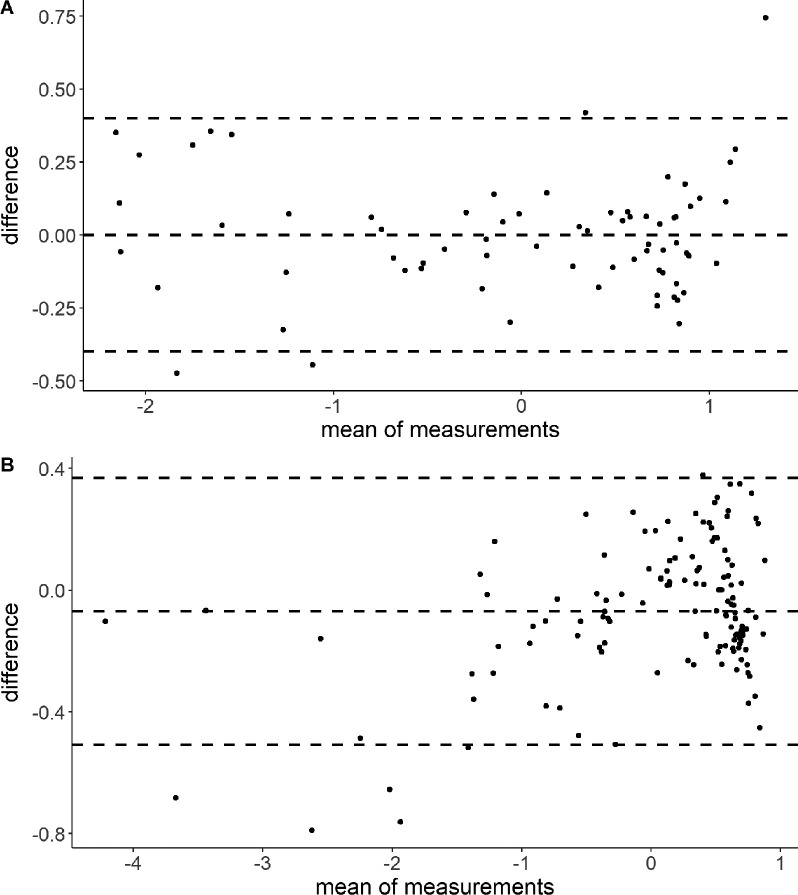
**Bland-Altman plot as a graphical representation of the agreement between the observed remaining time to culture conversion as the gold standard and the TB27 score with a limit of agreement of one standard deviation (upper and lower dashed line) in the validation cohort.** A) Consistency of TB27 and culture data in all samples of validation cohort. B) Consistency of TB27 and culture data in all measurements after the start of therapy and before the culture conversion of validation cohort.

The Bland-Altmann plot, which is a graphical tool for comparing 2 measurement methods, shows the difference between culture and TB27 and one standard deviation as limit of agreement above and below the mean in the validation cohort. In 7 measurements, the TB27 results deviated from the culture gold standard beyond the acceptable range, but 5 of these concerned the baseline measurement before the start of therapy (Figure 3).

**Figure 4.**
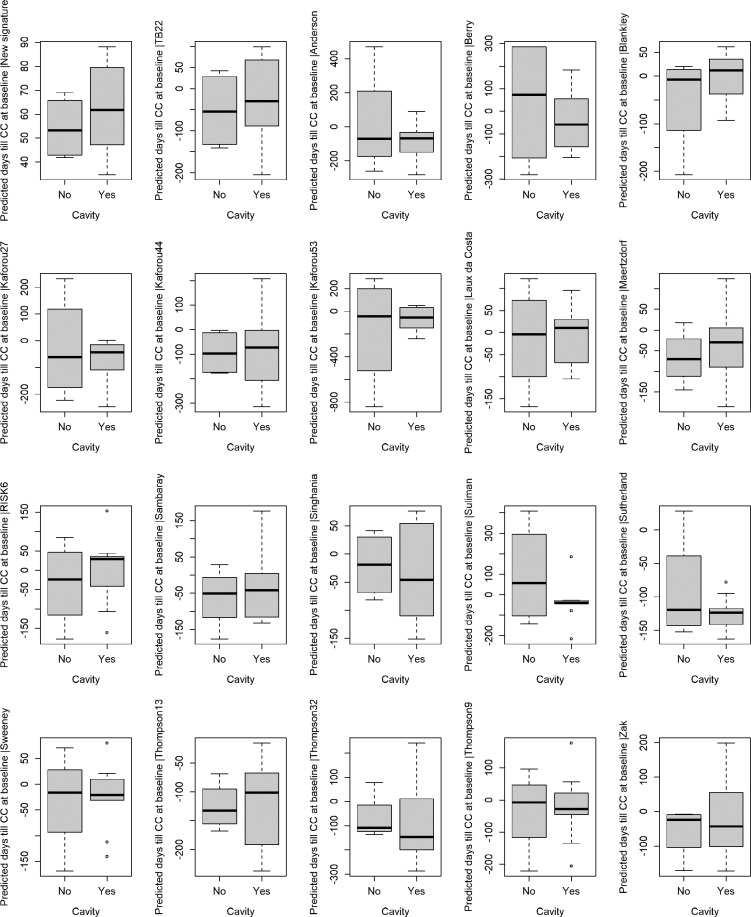
**Comparison of the 27-RNA gene signature model with published RNA-signatures and scores for predicting time left until culture conversion at baseline with consideration of presence or absence of cavities.** Y-axis: Predicted days left until culture conversion. Figure 4A: TB27. Figure 4B: TB22 [17]. Figure 4C: Anderson et al, 43 genes [18]. Figure 4D: Berry et al, 87 genes [19]. Figure 4E: Blankley et al, 4 genes [20]. Figure 4F: Kaforou et al, 27 genes [21]. Figure 4G: Kaforou et al, 44 genes [21]. Figure 4H: Kaforou et al, 53 genes [21]. Figure 4I: Laux da Costa et al, 3 genes [22]. Figure 4J: Maertzdorf et al, 3 genes [24]. Figure 4K: RISK6 genes [23]. Figure 4L: Sambarey et al, 10 genes [25]. Figure 4M: Singhania et al, 20 genes [26]. Figure 4N: Suliman et al, 4 genes [27]. Figure 4O: Sutherland et al, 4 genes [28]. Figure 4P: Sweeney et al, 3 genes [30]. Figure 4Q: Thompson et al, 16 genes [29]. Figure 4R: Thompson et al, 32 genes [29]. Figure 4S: Thompson et al, 9 genes [29]. Figure 4T: Zak et al, 16 genes [14].

The TB27 AI algorithm was compared to previously described signatures. TB27 was the only signature with a stringent prediction of time to culture conversion (Figure 2). Further, none of the signatures showed the difference between patients with and without cavities in terms of remaining TCC at the time of therapy initiation as clearly as TB27 (Figure 4A). Although other signatures tested also predicted shorter remaining times in the absence of cavities (Figure 4B-T), these also showed negative values at the start of therapy, which indicates that culture conversion had already occurred. Finally, the coefficient of determination R² as well as the correlation coefficient *r* were superior to those of previously described signatures (Table 2).

**Table 2.** Performance Comparison of Different TB-Associated RNA Signature Models for Predicting Time Left Until Culture Conversion

Signature	Number of genes	R^2^	Correlation coefficient
TB27	27	0.982	0.98
TB22 [17]	22	0.173	0.66
Anderson [18]	44	0.276	0.38
Berry [19]	83	0.348	0.44
Blankley [20]	4	0.187	0.60
Kaforou [21]	27	0.213	0.49
Kaforou [21]	44	0.323	0.214
Kaforou [21]	53	0.286	−0.01
Laux da Costa [22]	5	0.187	0.59
Maertzdorf [24]	3	0.137	0.50
RISK6 [23]	6	0.166	0.56
Sambarey [25]	10	0.104	0.38
Singhania [26]	20	0.201	0.54
Suliman [27]	4	0.200	0.43
Sutherland [28]	4	0.008	0.39
Sweeney [30]	3	0.169	0.61
Thompson [29]	9	0.167	0.51
Thompson [29]	16	0.069	0.39
Thomspon [29]	32	0.248	0.41
Zak [14]	16	0.229	0.47

## DISCUSSION

The point in time when *M. tuberculosis* becomes undetectable by culture-based methods during the course of anti-TB therapy is a critical milestone because subsequently the patient is no longer considered contagious, and further monitoring of the treatment response is significantly reduced. In this study, we identified and validated a TB-specific whole blood-based AI RNA-signature biomarker algorithm consisting of 27 genes to monitor treatment response by pathogen-free modeling of the time remaining to culture conversion. The algorithm was trained on a therapy monitoring variable and thus, allows conclusions about the therapy response.

Previous studies on treatment monitoring by RNA signatures focused on the discrimination between patients with good and poor treatment outcomes based on WHO definitions [32], or distinguished between active TB and latent *M. tuberculosis* infection or healthy controls [15]. Due to these indirect reference values, these signatures are primarily suitable for additional assessment of therapy response. Nevertheless, they cannot replace culture as a marker for viable bacteria. Another study also deals with therapy monitoring in connection with the end of therapy, but the score slopes are very low in the early therapy stage and are therefore not suitable as a monitoring tool in the first weeks of therapy [17].

The genes identified and validated in our work cover a broad spectrum of physiological and pathological functions. CD83, KIR2DS4, and IGHA1 show a direct immune system association [33–35], and guanylate-binding proteins (GBPs) notably model disease susceptibility and inflammatory processes [36]. This is specifically illustrated by the transcriptional differences found in the expression of GBPs in neutrophils matured by IFN-γ [37]. High concentrations of IGHA1 in plasma cells resulting from the activation of B cells by *M. tuberculosis* were also observed [35, 38]. CNTNAP3b correlates with CD8 T-cells with cytolytic activity to kill *M. tuberculosis*-infected cells via granule-mediated function [39, 40]. Upregulation of CYB5R1 has been found in TB-infected macrophages [41]. FAM20A has already been described in the context of TB-specific therapy response [17]. FCGR1B transcripts appear to have diagnostic relevance through correlation with bacterial load [42]. COL9A2 has previously been described to distinguish between LTBI and active TB [43].

A strength of this study is the relative size of n=149 of the identification cohort. Furthermore, the genetic information was compressed into scores to minimize overfitting. Finally, to the best of our knowledge, this is the first study to use culture data as a reference and thus, a direct surrogate for therapy monitoring. The weekly culture data of the patients in the German cohorts are, to our knowledge, also unique as a data basis.

This study also has certain limitations. We present findings of a single validation cohort. Further validation in a larger, independent cohort is necessary. Ideally, future prospective studies should be conducted to verify the practical prognostic applicability of the algorithm. The results presented here are based on a multi-step procedure, minimizing the risk of overfitting and increasing the likelihood of transferability to other patients. Furthermore, in comparison with other published TB signatures, TB27 showed the best fit with the time remaining until culture conversion and the presence of cavities.

Several RNA signatures have been developed and described specifically for different purposes in the context of tuberculosis, some of which have been used for comparison in this work. The algorithm described here has been developed for the dynamic evolution of the host response to the changing bacterial load during the course of therapy. It is important to note that the signatures used for comparison were designed for different purposes; in some cases not even for dynamic evolution but for differentiation between different disease states, outcomes, or similar. The different initial questions must be considered when evaluating the results of the comparisons. Based on this premise, it can also be assumed that the TB27 signature falls short of other signatures designed for other TB-specific questions, especially with regard to specific status differentiation. However, this work is based on the idea that a “one-signature-fits-all” approach neglects this contextual dependency, may result in lower specificity and sensitivity, and may miss important information about individual disease dynamics. Tailored signatures allow for more precise and clinically relevant decisions [44].

TB27 is an algorithm that represents a momentary impression of a dynamic development. In our cohorts, there were few cases of treatment failure, primarily characterised by prolonged time to culture conversion rather than relapse. In addition, relapses in both groups (MDR and non-MDR tuberculosis) may have been partially minimized by individual physician decisions to prolong treatment. The significance of this will need to be investigated in further studies.

In this study, only results from European cohorts were presented; the applicability to cohorts in other regions still needs to be assessed. The validation cohort was also significantly older than the patients in the identification cohort (*P*=0.036). Also, the proportion of patients with MDR-TB was significantly smaller in the validation cohort (n=16, 47.1%) than in the identification cohort (*P*=0.046). In addition, as a feasibility study, the cohorts did not include people living with HIV, children, or patients with solely extrapulmonary TB. Hence, broad applicability of our signature in certain patient groups must be verified in future studies. Notably the cohort of n=149 contributes to the robustness of the findings. RNA sequencing is currently a cost-intensive technology that requires trained personnel and can therefore only be used to a small extent in resource-limited settings. However, sequencing platforms and associated technologies are evolving rapidly, leading to increasing availability of high-throughput sequencing systems and advances in portable devices as well as bioinformatic processing, which could enable wider application even in resource-limited areas [45]. Furthermore, when using a gene signature based on 27 genes, targeted sequencing would be sufficient, which also leads to a better cost efficacy [46].

In conclusion, we identified and validated a whole blood-based AI RNA-biosignature algorithm containing 27 genes (TB27) that accurately predicts the remaining time until culture conversion during TB therapy. After further validation, this algorithm could become a valuable tool to monitor TB treatment responses and to predict non-contagiousness in individual patients. Technological advances in recent years have allowed a continued increase in RNA sequencing, opening up its future use in medical applications to a wider field, which also gives TB27 a chance of being useful in the medical treatment of TB patients.
